# Anastomotic stenosis following proximal gastrectomy with single flap valvulopasty successfully managed with endoscopic stricturotomy: a case report

**DOI:** 10.3389/fsurg.2023.1190301

**Published:** 2023-06-20

**Authors:** Yuan Tian, Qiankun Shao, Qiang Chen, Wei Peng, Rui Ren, Wei Gong, Tianhua Liu, Jianhong Zhu, Yongyou Wu

**Affiliations:** Department of Gastrointestinal Surgery, The Second Affiliated Hospital of Soochow University, Suzhou, China

**Keywords:** case report, adenocarcinoma of the esophagogastric junction (AEG), esophagogastric anastomotic stenosis, endoscopic stricturotomy, single flap technique

## Abstract

**Background:**

Due to its nutritional advantages over total gastrectomy, proximal gastrectomy (PG) with anti-reflux techniques has gained significant attention in East Asian countries in recent years. The double flap technique (DFT) and modified side overlap and fundoplication by Yamashita (mSOFY) are two promising anti-reflux interventions following PG. However, anastomotic stenosis after DFT and gastroesophageal reflux after mSOFY have been reported in several patients. To address these concerns, a hybrid reconstruction procedure was designed, namely, right-sided overlap with single flap valvulopasty (ROSF), for proximal gastrectomy, with the aim of reducing anastomotic stricture and reflux. Among the 38 patients who underwent ROSF at our hospital, one developed Stooler grade II anastomotic stenosis. Herein, we present the successful management of this patient through endoscopic stricturotomy (ES).

**Case summary:**

A 72-year-old female complaining of “epigastric pain and discomfort for more than 1 month” was diagnosed with adenocarcinoma of the esophagogastric junction (Siewert type II). She underwent laparoscopic-assisted PG and ROSF procedures at our hospital and recovered well after surgery. However, she started experiencing progressive difficulty in eating and vomiting approximately 3 weeks after the intervention. Endoscopy revealed Stooler grade II esophagogastric anastomotic stenosis. ES with insulated tip (IT) Knife nano was eventually performed, and the patient was able to resume a normal diet without experiencing any discomfort during the 5-month follow-up period.

**Conclusion:**

Endoscopic stricturotomy using IT Knife nano successfully treated anastomotic stenosis following ROSF with no associated complications. Thus, ES to treat anastomotic stenosis after PG with valvulopasty can be considered a safe option and should be performed in centers with the required expertise.

## Introduction

For Siewert type II adenocarcinoma of the esophagogastric junction (AEG), proximal gastrectomy (PG) with esophagogastrostomy and anti-reflux techniques are gaining widespread popularity in East Asian countries. Notably, double flap technique (DFT) and modified side overlap and fundoplication by Yamashita (mSOFY) are two representative reconstruction methods. However, anastomotic stenosis and esophageal reflux following DFT and mSOFY interventions have been reported in certain populations. Herein, we proposed a novel anti-reflux esophagogastrostomy method called right-sided overlap with single flap valvulopasty (ROSF). A right-opening single seromuscular flap and ROSF were conducted to improve blood supply to the flaps and reduce anastomotic stenosis as well as esophageal reflux (Table 1). Since March 1, 2021, we have performed the ROSF procedure in 38 cases without anastomotic leakage or symptomatic reflux esophagitis, with only one patient (2.6%) developing anastomotic stricture. In patients with postoperative anastomotic stenosis after DFT or mSOFY, balloon dilation (BD) is usually employed. However, BD may not always be effective for severe fibrotic stricture, necessitating repeated dilations in some instances.

**Table 1:** Comparison of anastomotic complications of ROSF and other esophagogastrostomies.

Types of reconstruction	Stenosis	Reflux esophagitis	Leakage
Esophagogastrostomy (1–12)	0%–52.2%	20%–65.2%	0%–18.2%
mSOFY (13, 14)	0%	7.1%	0%
DFT (9, 15–26)	0%–33.3%	0%–10.6%	0%–7.7%
ROSF^a^	2.6%	0%	0%

ROSF, right-sided overlap with single flap valvulopasty; mSOFY, modified side overlap and fundoplication by Yamashita; DFT, double flap technique.

^a^ From the follow-up data of 38 patients who underwent ROSF operation at our center (from March 2021 to October 2022). All patients were followed up at our outpatient department 1 week following discharge, 1 month after the operation, and 3 months after the operation. Additional visits were recommended whenever there were discomforts. All patients received barium meal gastroenterography. Twenty-two patients underwent gastroscopy for evaluation of reflux esophagitis. All patients were in regular telephone contact. In addition to endoscopy, our team used the GerdQ scale and the Gerd-HRQL scale for the diagnosis of reflux esophagitis and assessment of quality of survival.

In our case, we successfully performed endoscopic stricturotomy (ES) using the insulated tip (IT) Knife nano. Consequently, we present this case to discuss the rationale and potential application of ES in the treatment of anastomotic stenosis after esophagogastrostomy procedures with valvulopasty, such as DFT, mSOFY, and ROSF.

## Case presentation

**Figure 1:**
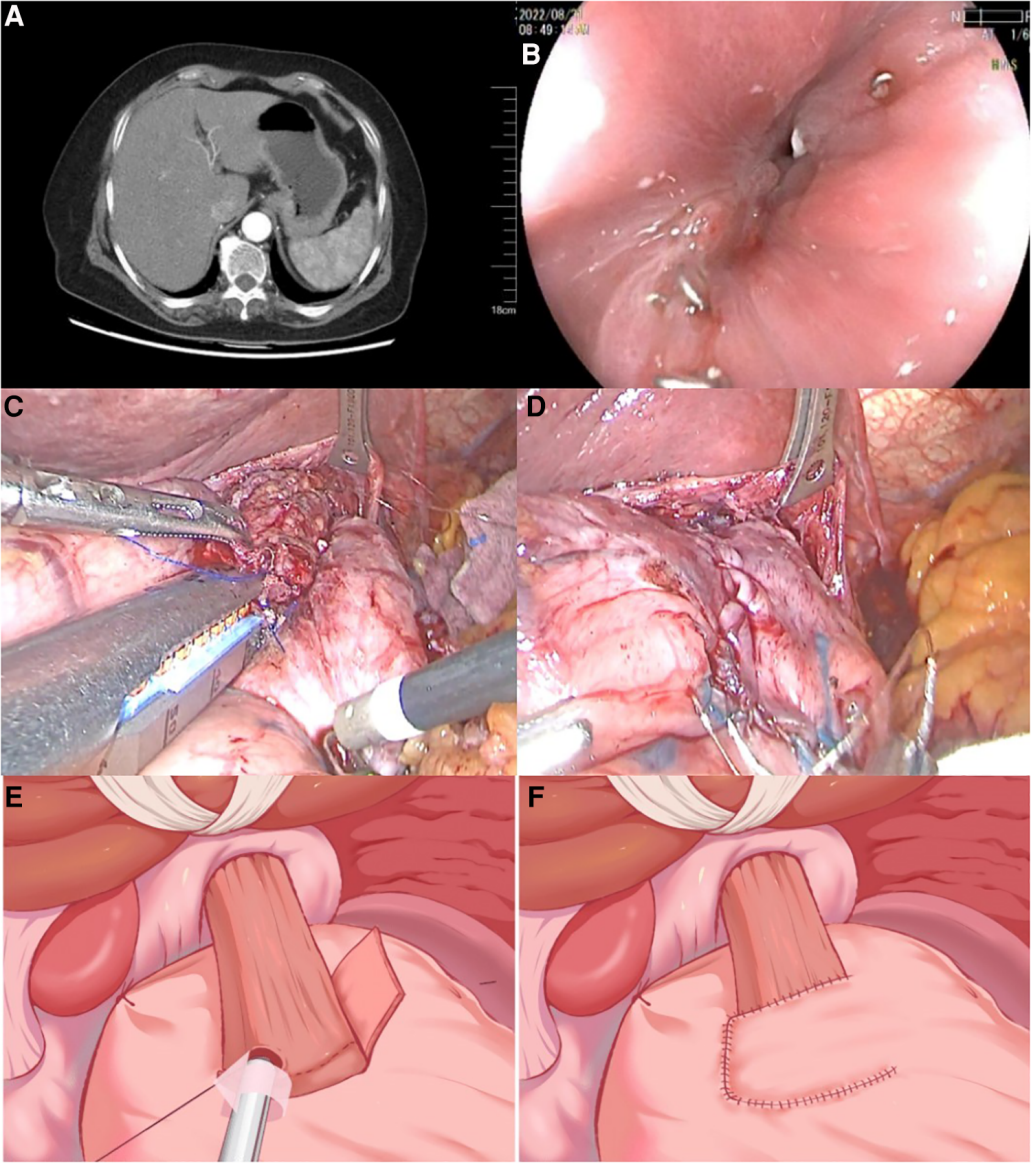
Preoperative findings. (**A**) Contrast-enhanced CT scan showed that the cardiac wall was thickened and enhanced. Small lymph node involvement was detected. (**B**) Gastroscopy confirmed the presence of a narrow anastomotic orifice between the esophagus and stomach, while the mucosa near the anastomotic orifice appeared smooth and intact. The diameter of the stenosis was about 3 mm. (**C,D**) Intraoperative pictures of ROSF surgery (side overlap anastomosis with linear stapler and muscle flap suture). (**E,F**) Schematic diagram illustrating the key steps of ROSF. CT, computed tomography; ROSF, right-sided overlap with single flap valvulopasty.

A 72-year-old female who presented with “epigastric pain and discomfort for more than 1 month” as the chief complaint was diagnosed with AEG (Siewert type II). Subsequently, the patient was referred to our hospital for surgical treatment. Preoperative computed tomography (CT) and gastroscopy confirmed AEG with the involvement of small perigastric lymph nodes (Figure 1A). The initial TNM staging was cT2N0-1M0. A laparoscopic PG with ROSF was thus performed. The patient experienced a smooth postoperative recovery and was discharged on the 8th day following surgery. One week after discharge, she resumed a normal diet without any issues. However, approximately 2 weeks later, she started experiencing progressive difficulty in eating accompanied by vomiting. Notably, she experienced no discomfort when switching to a liquid diet (Stooler grade II). As a result, she was readmitted for further management due to dysphagia 6 weeks following ROSF. Anastomotic stenosis was revealed through endoscopy (Figure 1B).

Generally, during the ROSF procedure, the overlap length between the esophagus and gastric mucosal is around 3 cm, providing enough room for conducting anastomosis. Therefore, the exact mechanism of stricture development in our patient is unclear. We surmised that staple line adhesion could have caused the anastomotic stenosis. Given that attempts at dilation using a gastroscope proved unsuccessful due to the presence of fibrotic scar tissue, ES with IT Knife nano was attempted. The posterior wall of the anastomosis was chosen as a safe area for incision, as it was located behind the posterior wall adjacent to the pseudo-gastric fundus. To prevent reflux, an incision of approximately 1 cm was made on the left lateral-posterior side of the anastomosis, ensuring a spacious opening for gastroscope insertion. Following the intervention, proton pump inhibitor (PPI) was administered for 2 weeks, and the patient recovered well. During the 5-month follow-up period, no symptoms of dysphagia or reflux were reported. A gastroscopy re-examination revealed no evidence of stricture, esophagitis, or inflammation at the anastomotic site. Additionally, the patient's body weight increased following the ES procedure, and laboratory tests showed that nutritional parameters were within the normal range.

## Treatment

**Figure 2:**
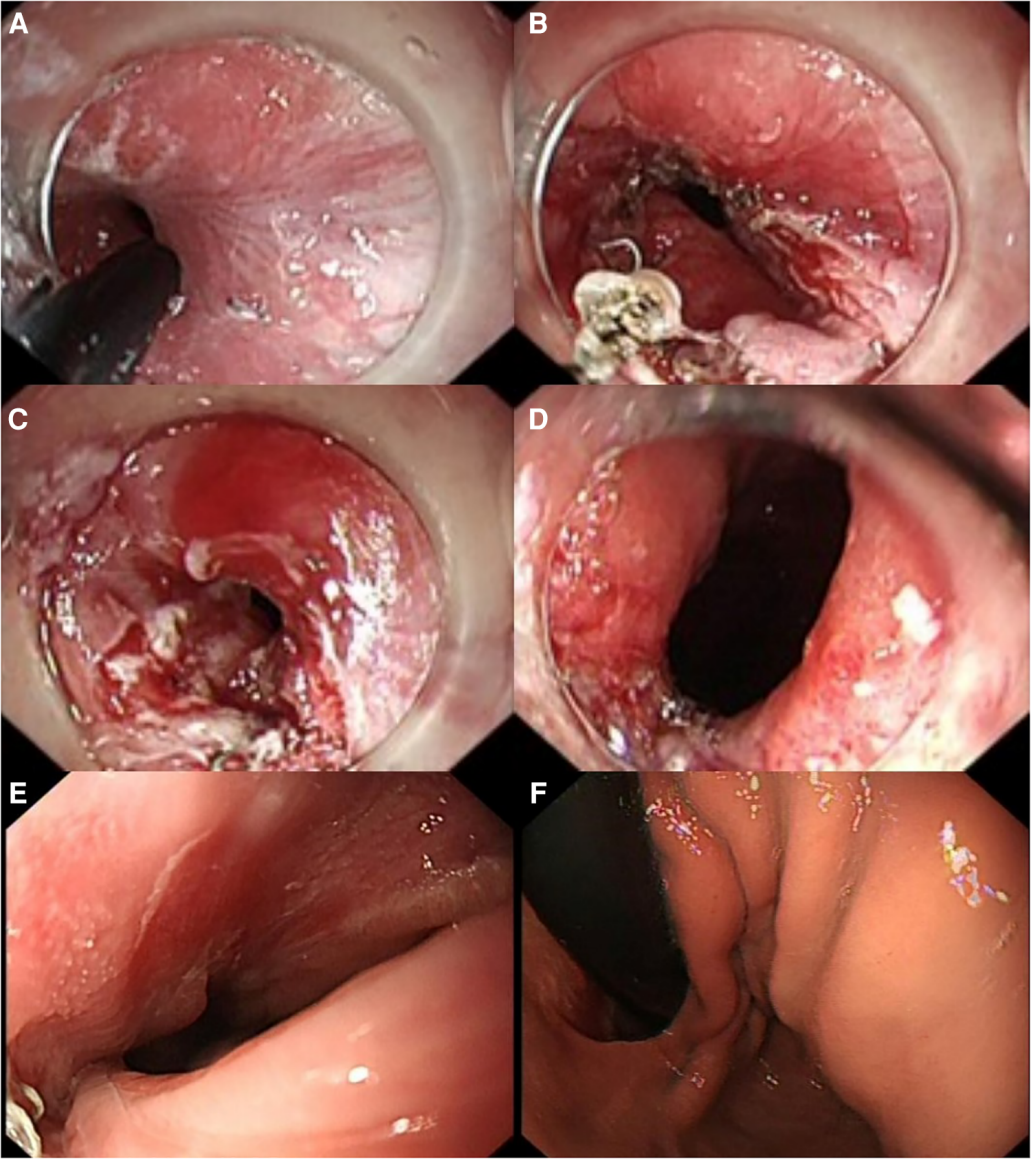
Endoscopic incision procedure and gastroscopy images (2 months after ES). (**A,B**) IT Knife nano was used to make an incision along the anastomotic staple line from the stenosis to the left side, gradually extending the stenosis. (**C,D**) Upon retracting the gastroscope, the relaxation and contraction of the cardia were observed, indicating good movement without any obstruction hindering re-entry of the gastroscope. (**E**) No anastomotic stenosis was seen on gastroscopy. (**F**) Anatomical structures of the cardia, such as the pseudo-gastric fundus and mucosal folds, could be seen after the gastroscope passed through the anastomosis. ES, endoscopic stricturotomy.

The treatment procedure is shown in Figure 2.

## Outcome and follow-up

The nutritional parameters during follow-up are shown in Table 2, and the gastroscopy images reviewed 2 months after ES are shown in Figures 2E,F. The patient resumed normal diet without discomfort until January 2023.

**Table 2:** Nutritional parameters.

		2022/7/16 (Pre-ROSF)	2022/7/21	2022/7/23	2022/7/26	2022/9/10 (Post-ES)	2022/9/12	2022/11/12	2022/11/18
Laboratory tests	Hemoglobin (g/L)	137	142	131	131	160	132	127	129
	Triglyceride (mmol/L)	2.01	1.21	1.23	1.15	1.57	3.87	3.71	3.72
	Albumin (g/L)	45.8	37.8	42.4	39.4	51.7	41.7	46.1	44.5
	Prealbumin (g/L)	0.26	0.19	0.15	0.21	0.18	0.15	0.22	0.21
	Total protein (g/L)	76.1	66.4	67.9	70.9	84.9	67.0	77.2	74.2
Weight (kg)		51.2	48.7	49	49.1	44.7	44.9	47.5	47.8
BMI (kg/m^2^)		21.3	20.2	20.4	20.4	18.6	18.6	19.7	19.9

ROSF, right-sided overlap with single flap valvulopasty; ES, endoscopic stricturotomy.

## Discussion

Esophagogastric anastomotic stenosis is a relatively common complication after PG, and its management is challenging. Endoscopic intervention is typically the standard approach, encompassing balloon dilation, incision, and stent placement, with or without glucocorticosteroid injection to reduce the inflammatory response and prevent restenosis (27). Endoscopic balloon dilation is typically preferred since it is less invasive, safer, and associated with fewer complications, albeit with the potential for recurrence and the need for multiple dilation sessions. On the other hand, endoscopic incision (including ES) is more suitable for patients with severe stenosis or complex refractory stenosis (27, 28). Studies reported no significant difference between its efficacy and balloon dilation in terms of first-time outcome (28, 29). Nevertheless, the risk of postoperative complications may be slightly increased (28). Based on the characteristics of ROSF, we sought to explore an endoscopic incision technique capable of alleviating stenosis in a single treatment. In cases of anastomotic stenosis in side-to-side anastomoses between the esophagus and the anterior wall of the stomach using a linear stapler, a lateral incision along the staple line on the left side of the anastomosis is the best approach. Incision toward the posterior wall of the esophagus is also safe but may result in a diminished anti-reflux effect. Anastomotic stenosis after esophagogastrostomy with valvulopasty, including DFT, mSOFY, and ROSF procedures, can be managed by ES, but the site, direction, and size of the incision should be meticulously planned by taking into account the specific details of the previous valvulopasty procedure.

Previous studies have demonstrated a correlation between the occurrence of esophagogastric anastomotic stenosis and scar formation at the anastomosis site, with connective tissue growth factor (CTGF) and transforming growth factor-β1 (TGF-β1) playing significant roles in tissue scar formation (30). Importantly, factors such as gastric acid stimulation and postoperative fasting may exacerbate anastomotic fibroplasia and stenosis formation. In addition to the endoscopic injection of hormones, the use of PPI has been shown to effectively reduce gastric acid irritation and fibrotic scar formation. Notably, diet is also an important factor in preventing esophagogastric anastomotic stenosis. Given the favorable safety profile of ROSF, we recommend a quick transition from a liquid diet to a normal diet postoperatively. Firm-textured foods naturally exert a dilating effect on the anastomosis, which helps prevent stenosis formation. Nonetheless, patients should still adhere to a balanced diet to avoid restenosis after undergoing ES. Of note, an excessively conservative approach to diet transition may instead contribute to stenosis. The utilization of diet as a physical dilation method, similar to balloon dilation, thus warrants further exploration and study.

## Conclusion

Endoscopic stricturotomy using IT Knife nano successfully treated anastomotic stenosis following ROSF with no associated complications. Therefore, utilizing ES to treat anastomotic stenosis after PG with valvulopasty can be considered a safe option and should be performed in centers with the required expertise.

## Data Availability

The original contributions presented in the study are included in the article, further inquiries can be directed to the corresponding authors.

## References

[1] Ahn SH Lee JH Park DJ Kim HH. Comparative study of clinical outcomes between laparoscopy-assisted proximal gastrectomy (LAPG) and laparoscopy-assisted total gastrectomy (LATG) for proximal gastric cancer. Gastric Cancer. (2013) 16(3):282–9. 10.1007/s10120-012-0178-x 22821182

[2] Sakuramoto S Yamashita K Kikuchi S Futawatari N Katada N Moriya H Clinical experience of laparoscopy-assisted proximal gastrectomy with Toupet-like partial fundoplication in early gastric cancer for preventing reflux esophagitis. J Am Coll Surg. (2009) 209(3):344–51. 10.1016/j.jamcollsurg.2009.04.011 19717038

[3] Seshimo A Miyake K Amano K Aratake K Kameoka S. Clinical outcome of esophagogastrostomy after proximal gastrectomy for gastric cancer. Hepatogastroenterology. (2013) 60(123):616–9. 10.5754/hge12782 23108089

[4] Masuzawa T Takiguchi S Hirao M Imamura H Kimura Y Fujita J Comparison of perioperative and long-term outcomes of total and proximal gastrectomy for early gastric cancer: a multi-institutional retrospective study. World J Surg. (2014) 38(5):1100–6. 10.1007/s00268-013-2370-5 24310733

[5] Tokunaga M Ohyama S Hiki N Hoshino E Nunobe S Fukunaga T Endoscopic evaluation of reflux esophagitis after proximal gastrectomy: comparison between esophagogastric anastomosis and jejunal interposition. World J Surg. (2008) 32(7):1473–7. 10.1007/s00268-007-9459-7 18264827

[6] Chen XF Zhang B Chen ZX Hu JK Dai B Wang F Gastric tube reconstruction reduces postoperative gastroesophageal reflux in adenocarcinoma of esophagogastric junction. Dig Dis Sci. (2012) 57(3):738–45. 10.1007/s10620-011-1920-7 21953142

[7] Nakamura M Nakamori M Ojima T Katsuda M Iida T Hayata K Reconstruction after proximal gastrectomy for early gastric cancer in the upper third of the stomach: an analysis of our 13-year experience. Surgery. (2014) 156(1):57–63. 10.1016/j.surg.2014.02.015 24799083

[8] Chen S Li J Liu H Zeng J Yang G Wang J Esophagogastrostomy plus gastrojejunostomy: a novel reconstruction procedure after curative resection for proximal gastric cancer. J Gastrointest Surg. (2014) 18(3):497–504. 10.1007/s11605-013-2391-2 24163139

[9] Saze Z Kase K Nakano H Yamauchi N Kaneta A Watanabe Y Functional benefits of the double flap technique after proximal gastrectomy for gastric cancer. BMC Surg. (2021) 21(1):392. 10.1186/s12893-021-01390-1 34740344 PMC8569978

[10] Hong J Qian L Wang YP Wang J Hua LC Hao HK. A novel method of delta-shaped intracorporeal double-tract reconstruction in totally laparoscopic proximal gastrectomy. Surg Endosc. (2016) 30(6):2396–403. 10.1007/s00464-015-4490-5 26416371

[11] Adachi Y Inoue T Hagino Y Shiraishi N Shimoda K Kitano S. Surgical results of proximal gastrectomy for early-stage gastric cancer: jejunal interposition and gastric tube reconstruction. Gastric Cancer. (1999) 2(1):40–5. 10.1007/s101200050019 11957069

[12] Aburatani T Kojima K Otsuki S Murase H Okuno K Gokita K Double-tract reconstruction after laparoscopic proximal gastrectomy using detachable ENDO-PSD. Surg Endosc. (2017) 31(11):4848–56. 10.1007/s00464-017-5539-4 28389804

[13] Yamashita Y Yamamoto A Tamamori Y Yoshii M Nishiguchi Y. Side overlap esophagogastrostomy to prevent reflux after proximal gastrectomy. Gastric Cancer. (2017) 20(4):728–35. 10.1007/s10120-016-0674-5 27942874

[14] Yamashita Y Tatsubayashi T Okumura K Miyamoto T Ueno K. Modified side overlap esophagogastrostomy after laparoscopic proximal gastrectomy. Ann Gastroenterol Surg. (2022) 6(4):594–9. 10.1002/ags3.12549 35847432 PMC9271030

[15] Kumamoto T Sasako M Ishida Y Kurahashi Y Shinohara H. Clinical outcomes of proximal gastrectomy for gastric cancer: a comparison between the double-flap technique and jejunal interposition. PLoS One. (2021) 16(2):e0247636. 10.1371/journal.pone.0247636 33626086 PMC7904176

[16] Hayami M Hiki N Nunobe S Mine S Ohashi M Kumagai K Clinical outcomes and evaluation of laparoscopic proximal gastrectomy with double-flap technique for early gastric cancer in the upper third of the stomach. Ann Surg Oncol. (2017) 24(6):1635–42. 10.1245/s10434-017-5782-x 28130623

[17] Kuroda S Nishizaki M Kikuchi S Noma K Tanabe S Kagawa S Double-flap technique as an anti-reflux procedure in esophagogastrostomy after proximal gastrectomy. J Am Coll Surg. (2016) 223(2):e7–13. 10.1016/j.jamcollsurg.2016.04.041 27157920

[18] Hosoda K Washio M Mieno H Moriya H Ema A Ushiku H Comparison of double-flap and OrVil techniques of laparoscopy-assisted proximal gastrectomy in preventing gastroesophageal reflux: a retrospective cohort study. Langenbecks Arch Surg. (2019) 404(1):81–91. 10.1007/s00423-018-1743-5 30612151

[19] Muraoka A Kobayashi M Kokudo Y. Laparoscopy-assisted proximal gastrectomy with the hinged double flap method. World J Surg. (2016) 40(10):2419–24. 10.1007/s00268-016-3510-5 27094564

[20] Kuroda S Choda Y Otsuka S Ueyama S Tanaka N Muraoka A Multicenter retrospective study to evaluate the efficacy and safety of the double-flap technique as anti-reflux esophagogastrostomy after proximal gastrectomy (rD-FLAP study). Ann Gastroenterol Surg. (2018) 3(1):96–103. 10.1002/ags3.12216 30697614 PMC6345660

[21] Tanaka Y Isobe T Fujita F Sudo T Kaku H Minami T The benefits of a double-flap technique after proximal gastrectomy in upper-third gastric cancer. Jpn J Gastroenterol Surg. (2019) :498. 10.5833/jjgs.2018.0143

[22] Long VD Hai NV Thong DQ Dat TQ Quoc HLM Minh TA Clinical outcomes of laparoscopic proximal gastrectomy with double-flap reconstruction for tumors in the upper third of the stomach. Surg Laparosc Endosc Percutan Tech. (2022) 32(3):409–14. 10.1097/SLE.0000000000001053 35583586

[23] Yu B Park KB Park JY Lee SS Kwon OK Chung HY Double tract reconstruction versus double flap technique: short-term clinical outcomes after laparoscopic proximal gastrectomy for early gastric cancer. Surg Endosc. (2022) 36(7):5243–56. 10.1007/s00464-021-08902-3 34997340

[24] Kikuchi S Nemoto Y Katada N Sakuramoto S Kobayashi N Shimao H Results of follow-up endoscopy in patients who underwent proximal gastrectomy with jejunal interposition for gastric cancer. Hepatogastroenterology. (2007) 54(73):304–7. PMID: . 17419280

[25] Takayama Y Kaneoka Y Maeda A Fukami Y Onoe S. Comparison of outcomes of laparoscopy-assisted and open proximal gastrectomy with jejunal interposition for early gastric cancer in the upper third of the stomach: a retrospective observational study. Asian J Endosc Surg. (2018) 11(4):329–36. 10.1111/ases.12469 29570950

[26] Shaibu Z Chen Z Mzee SAS Theophilus A Danbala IA. Effects of reconstruction techniques after proximal gastrectomy: a systematic review and meta-analysis. World J Surg Oncol. (2020) 18(1):171. 10.1186/s12957-020-01936-2 32677956 PMC7367236

[27] Beilstein MC Kochman ML. Endoscopic incision of a refractory esophageal stricture: novel management with an endoscopic scissors. Gastrointest Endosc. (2005) 61(4):623–5. 10.1016/s0016-5107(04)02787-7 15812426

[28] Hordijk ML van Hooft JE Hansen BE Fockens P Kuipers EJ. A randomized comparison of electrocautery incision with savary bougienage for relief of anastomotic gastroesophageal strictures. Gastrointest Endosc. (2009) 70(5):849–55. 10.1016/j.gie.2009.02.023 19573869

[29] Sami SS Haboubi HN Ang Y Boger P Bhandari P de Caestecker J UK guidelines on oesophageal dilatation in clinical practice. Gut. (2018) 67(6):1000–23. 10.1136/gutjnl-2017-315414 29478034 PMC5969363

[30] Zhao H Zhao L Zhou Z Wu Y. The roles of connective tissue growth factor in the development of anastomotic esophageal strictures. Arch Med Sci. (2015) 11(4):770–8. 10.5114/aoms.2015.48147 26322089 PMC4548024

